# Comparison of different protocols for demineralization of cortical bone

**DOI:** 10.1038/s41598-021-86257-4

**Published:** 2021-03-29

**Authors:** Siyuan Pang, Frances Y. Su, Amesha Green, Justin Salim, Joanna McKittrick, Iwona Jasiuk

**Affiliations:** 1 Department of Mechanical Science and Engineering, University of Illinois at Urbana Champaign, 1206 West Green Street, Urbana, IL 61801 USA; 2 Department of Mechanical and Aerospace Engineering and Materials Science and Engineering Program, University of California, San Diego, 9500 Gilman Dr, La Jolla, CA 92093-0411 USA; 3 Department of Chemical, Biological, and Bio Engineering, North Carolina Agricultural and Technical State University, 1601 E Market St, Greensboro, NC 27401 USA; 4 Department of Bioengineering, University of California, San Diego, 9500 Gilman Dr., La Jolla, CA 92093-0412 USA

**Keywords:** Analytical biochemistry, Biophysical methods, Microscopy, Structure determination, Biochemistry, Biological techniques, Biotechnology, Chemical biology, Structural biology, Medical research, Materials science

## Abstract

Bone is a biological composite material consisting of two main components: collagen and mineral. Collagen is the most abundant protein in vertebrates, which makes it of high clinical and scientific interest. In this paper, we compare the composition and structure of cortical bone demineralized using several protocols: ethylene-diamine-tetraacetic acid (EDTA), formic acid (CH_2_O_2_), hydrochloric acid (HCl), and HCl/EDTA mixture. The efficiencies of these four agents were investigated by assessing the remaining mineral quantities and collagen integrity with various experimental techniques. Raman spectroscopy results show that the bone demineralized by the CH_2_O_2_ agent has highest collagen quality parameter. The HCl/EDTA mixture removes the most mineral, but it affects the collagen secondary structure as amide II bands are shifted as observed by Fourier transform infrared spectroscopy. Thermogravimetric analysis reveals that HCl and EDTA are most effective in removing the mineral with bulk measurements. In summary, we conclude that HCl best demineralizes bone, leaving the well-preserved collagen structure in the shortest time. These findings guide on the best demineralization protocol to obtain high-quality collagen from bone for clinical and scientific applications.

## Introduction

Bone is a biological material with a complex composite structure that gives bone the high strength, toughness, and lightweight required for its mechanical functions^1^. The structural organization of bone has several hierarchical levels^2,3^. The macroscale (larger than 10 mm) represents the whole bone, while the mesoscale (~ 500 µm to 10 mm) includes cancellous (spongy) and cortical (compact) bones. The microscale (~ 10 µm to 500 µm) consists of cylindrical osteons (cortical bone) and crescent-shaped trabeculae (cancellous bone) which at the sub-microscale (~ 1–10 µm) are made of lamellae. At the nanoscale, lamellae contain collagen fibrils (~ 50–100 nm in diameter) mineralized with intra- and extra-fibrillar hydroxyapatite crystals (~ 50 × 25 × 3 nm^3^). Bone is made of organic components (90 volume percent (vol%) type I collagen and 10 vol% non-collagen proteins and other organics), an inorganic phase (mineral), and water, which occupy around 40, 40, and 20 vol% of bone, respectively^4^.

Collagen is the most abundant protein in mammals and is thus of high scientific, archaeological, and clinical interests. Bone structure can be studied by comparing untreated and demineralized bone (e.g.,^5^) to elucidate the role of collagen on its mechanical properties. In archaeological studies, collagen isolated from bone is used for radiocarbon dating (e.g.,^6^). Clinically, collagen has been used for tissue constructs and scaffolds for humans^7,8^ and animals^9,10^. Bone is usually first ground into particles ranging from 100 to 500 µm or cut into other small units to produce demineralized bone matrix (DBM). However, it is still not well understood how demineralization methods affect collagen, and thus, more studies are needed on how to obtain high-quality DBM for various medical applications. Such studies can also provide further insights into the collagen architecture in bone, collagen properties, and collagen’s contributions to bone’s mechanical properties.

Demineralization agents such as ethylene-diamine-tetraacetic acid (EDTA), hydrochloric acid (HCl), formic acid (CH_2_O_2_), and citric acid have been widely employed to obtain isolated collagen. The reaction rate, demineralization efficiency, and the effect on residual collagen were addressed in previous studies comparing different bone demineralization agents. Frank et al.^11^ compared cellular components but did not investigate the collagen structure. Amaral et al.^12^ studied the demineralized bone with scanning electron microscopy (SEM) but did not quantitatively analyze bone composition. Guibas et al.^13^ compared the efficiencies of mineral removal with shorter and longer treatment times using different protocols but did not analyze the structural integrity of collagen. Among previous studies, EDTA^14,15^ and HCl^5,16^ are the most common agents used for demineralization. Those studies concluded that in contrast to EDTA^17,18^, HCl alters collagen morphology. A comparison of 0.1 M EDTA and 0.6 M HCl revealed that EDTA resulted in an almost intact, hierarchically ordered collagen structure, while the hydrolysis induced by HCl destroyed the collagen structure^17^. Park et al.^19^ also investigated the HCl treatment time and showed that inorganic components and crystallinity sharply decreased as treatment time increased. However, a 60-min treatment time still could not eliminate all the mineral. Several studies used CH_2_O_2_ as a demineralization agent^11^ and found it to be more efficient at demineralization than EDTA since it requires less time and has equal efficacy as EDTA^20^. A recently proposed solution, called ETDA (HCl/EDTA mixture), is a mixture of 12 weight percent (wt%) HCl (3.3 M), 0.07 wt% EDTA (2.4 mM), 0.014 wt% sodium tartrate (C_4_H_4_O_6_Na_2_) (0.72 mM), and 0.8 wt% potassium tartrate (KNaC_4_H_4_O_6_·4H_2_O) (28 mM) in water^21^. The ETDA mixture performed similarly to EDTA, but the treatment time was much shorter. However, the collagen integrity after the ETDA mixture treatment was only determined by observing the collagen morphology. Thus, a more quantitative study should be conducted to investigate the efficiency of this method. So far, comparison studies have used a formalin-fixed bone as a starting material since most comparison studies use demineralization for histology sample preparation. This fixation affects not only the chemical but also the mechanical properties of demineralized bone. Thus, the most effective bone demineralization protocol is still not well established.

This paper presents a comprehensive comparison of demineralization methods using unfixed bone. Important metrics for an effective bone demineralization protocol are the efficiency of the mineral removal and the integrity of the remaining collagen. Micro-porosity of demineralized bone is not addressed in this paper since previous studies have shown through histology and SEM that the microstructure is preserved under demineralization protocols^22,23^. The results of this study will serve as a framework for future investigations of mechanical properties and biocompatibility of bone-derived collagen.

This paper addresses the effectiveness of four demineralization agents: EDTA, HCl, CH_2_O_2_, and ETDA (HCl/EDTA mixture) in removing mineral from bone and their influence on collagen structure. Several characterization techniques were employed: Raman spectroscopy, Fourier transform infrared spectroscopy (FTIR) spectroscopy, thermogravimetric analysis (TGA), and SEM. This study’s objective was to identify the best demineralization protocols to obtain high-quality collagen from bone for scientific studies and medical applications.

## Materials and methods

### Sample preparation

Femurs from 6-month-old pigs were obtained from the Meat Science Lab at the University of Illinois at Urbana-Champaign and Animal Technologies, Inc. (Tyler, TX, USA). Cortical bone was cut into dimensions of 2 mm × 2 mm × 4 mm from the mid-diaphysis section along the longitudinal direction using a diamond blade sectioning saw (Isomet 1000, Buehler, USA), and smoothed using 1200 grit sandpaper. The samples were then submerged into 20 mL solutions at room temperature of either 1 M EDTA (E9884, Sigma-Aldrich, St. Louis, MO, USA), 0.5 M CH_2_O_2_ (ACROS Organics, New Jersey, USA), 0.5 M HCl (EMD Millipore Corporation, Billerica, MA, USA), or the HCl/EDTA mixture. The samples were placed on a rocker to ensure agitation, and solutions were changed each day. Specimens submerged in HCl, CH_2_O_2_, and the HCl/EDTA mixture were treated until they were translucent when observed through stereomicroscopy, defined as the endpoint. EDTA-treated samples did not become transparent, so these samples were weighed to determine the endpoint. To study the weight loss, samples were removed from the solution, dabbed dry with a tissue, and weighed. If the sample’s weight decreased, the solution was changed, and the sample was treated for another day. Weights were recorded before and during the EDTA treatment. While the acid-demineralized samples lose weight during treatment, the weight-loss method cannot be used reliably due to collagen’s degradation in acidic solutions^23^, which causes the samples to continue to lose weight even after they have been fully demineralized. After each treatment, samples were placed in a sonicator for 15 min with deionized water to rinse residual chemicals. The samples were rinsed five times for five minutes each time. Table 1 summarizes the sample preparation methods.

**Table 1:** Sample preparation by demineralization agents: EDTA (ethylene-diamine tetraacetic acid), formic acid (CH_2_O_2_), hydrogen chloride (HCl), and HCl/EDTA mixture.

Agents	Concentration	Treatment time	Procedures
Untreated	N/A	N/A	Cut samples to 2 mm × 2 mm × 4 mm; polish; demineralization treatments on an agitation rocker until the samples were translucent (except for EDTA mixture—endpoint was based on the weighing method; remove chemicals by a sonicator; rinse samples by deionized water; dehydration by ethanol; critical point drying
EDTA	1 M	7 days	
CH_2_O_2_	0.5 M	2 days	
HCl	0.5 M	7 h	
HCl/EDTA mixture	3.3 M HCl, 2.4 mM EDTA, 0.72 mM C_4_H_4_O_6_Na_2_, 28 mM KNaC_4_H_4_O_6_·4H_2_O	3 h	

### Raman spectroscopy

The samples prepared for Raman spectroscopy were dehydrated in water/ethanol solutions where the ethanol concentration was gradually elevated from 25%, 50%, 75%, to 100 vol. %, for five minutes each. After being critical point dried (Autosamdri-931 Series Supercritical Point Dryer, Tousimis, Rockville, MD, USA), samples were then analyzed with a Raman spectrometer (NanoPhoton Raman 11, Osaka, Japan). A 785 nm laser with 1 mW power for 100 s of exposure was employed, and a 20 $\times$/0.45 objective was used with line-imaging infrared for scanning. Each sample had a spectrum from a 400 μm line-scanning that averaged 400 dot-scanning spectra. Three spectra were obtained for each treatment. In the reported data, we did not separate the pores data. A line-scanning may cross several pores on sample surface. We took three measurements to capture a bulk composition and minimize the effect of pores. Lateral spacing resolution can be calculated using the formula 0.51 λ/NA = 0.9 µm, where λ is a wavelength of the laser and NA is a numerical aperture^25^. To estimate the probing depth, we assumed bone tissue to be transparent and its absorption coefficient µ_a_ at 785 nm wavelength to be around 0.012 by interpolation of skull data according to the study by Cassano et al.^24^. The axial resolution, which is in the direction of light propagation, was determined based on the study of Cole et al*.*^25^. The axial resolution = 0.88 λ/(n – sqrt(n^2^ – NA^2^)) = 6.5 μm, where n is the refractive index of the immersion medium. The baseline drift due to background fluorescence was subtracted using a cubic spline fit.

The effectiveness of the demineralization chemicals was quantitatively assessed by calculating several parameters^26^. The mineral-to-matrix ratio, which is the intensity ratio of the primary phosphate band *v*_1_PO at 595 cm^−1^ to the amide III band at 1250 cm^−1^ is a measurement of the degree of mineralization. Amide III band is used to represent the matrix in mineral-to-matrix ratio since amide III band is less sensitive to the polarization of Raman laser light. The mineral crystallinity is expressed by the inverse of the full-width-at-half-maximum (1/FWHM) of the *v*_1_PO band. FWHM of a spectrum in Raman shows the structural distribution. Crystalline materials have narrower peaks than amorphous materials. Crystallinity reflects bone crystal size and lattice perfection^27^. The larger the value of 1/FWHM, the more ordered the mineral crystals are, and the longer the crystal c-axis length is^28^. The collagen quality parameter that describes collagen’s secondary structure and level of cross-links’ rupture, defined as the band area ratio of 1660 cm^−1^ to 1690 cm^−1^ under the amide I envelope^29^, was also determined.

### Fourier-transform infrared spectroscopy

FTIR was performed in addition to Raman spectroscopy. The absorption of infrared light in FTIR induces molecular vibrations. The molecular bonds within materials can be determined by measuring the vibrational frequency. For the Raman spectroscopy, a laser light source is scattered by molecules at different wavelengths^29^. Both FTIR and Raman spectroscopies should be performed to complement and validate results since some functional groups that cannot be detected by one technique can be measured by the other. FTIR samples were prepared using the method described in “Raman Spectroscopy”. The spectra were collected in the absorbance mode in the air with ~ 25 N force applied to the specimen measurement surface using an ATR Spectrum Two (PerkinElmer, Waltham, MA, USA) in attenuated total reflection (ATR) mode. A background spectrum was first taken every thirty minutes to ensure accurate background subtraction from the spectra using Spectrum 10™ software (PerkinElmer, Waltham, MA, USA). The baseline of each spectrum was corrected by the same methods as for Raman spectroscopy.

### Scanning electron microscopy

After demineralization treatments were performed, samples were fixed with neutral buffered formalin for one day to prevent sample degradation. Specimens were then rinsed and dehydrated using the methods described in “Raman Spectroscopy”. Subsequently, samples were embedded in epoxy (Epoxicure 2 Resin and Hardener, Buehler, USA), and the surfaces were smoothed with a Leica Ultracut UCT ultramicrotome (Leica Biosystems, Wetzlar, Germany) using a glass knife. Specimens were sputtered with iridium in an Emitech K575X (Quorum Technologies Ltd, East Sussex, UK). A field emission SEM (ZEISS Sigma 500, Carl Zeiss AG, Oberkochen, Germany) was employed to image the collagen structure. The voltage range used was between 1 and 3 kV.

Energy dispersive X-ray spectroscopy (EDS) was performed on each sample using the IXRF Iridium Ultra (IXRF Systems, Inc., Austin, TX, USA) at 20 kV to measure the atomic percentages and weight percentages of the main elements in bone: carbon (C), nitrogen (N), oxygen (O), phosphorus (P), calcium (Ca), and sodium (Na). Iridium Ultra uses the ZAF algorithm to calculate mass and atomic percentages.

### Thermogravimetric analysis

Specimens were dehydrated using the same methods as described in “Raman Spectroscopy”. After dehydration, samples were heated from room temperature to 800 ˚C at 10 ˚C/min in a TGA instrument (Pyris 1, PerkinElmer, Waltham, MA, USA). For bone, 250 ˚C is the point where all free and bound water is evaporated, and collagen decomposition and combustion take place from 250 to 750 ˚C^30^. The remaining solid after 750 ˚C is the mineral.

### Statistical method

Three bone samples from each treatment (untreated, EDTA-, CH_2_O_2_-, HCl-, HCl/EDTA mixture-treated) were imaged using Raman spectroscopy, FTIR, and SEM. Two samples from each treatment were tested by TGA. One-way ANOVA using OriginLab (OriginLab 2019, Northampton, USA) was performed to examine a statistical significance (*p* < 0.05) between different treatments. Error bars in plots were calculated based on standard deviation.

## Results and discussion

Endpoints of demineralization (sample transparency or end of weight loss) were different for each chemical treatment. The transparency method was used for acid treatments since it was shown to give similar results as an x-ray method, which is considered the most accurate method for endpoint determination^31^. For strong acid solutions, which include the HCl/EDTA mixture and HCl, the endpoints were reached at approximately three and seven hours, respectively. The CH_2_O_2_-treated samples became transparent after approximately two days of treatment. Weights of the EDTA-treated samples were measured before and during treatment. Compared to the weights of untreated samples, the weights of treated samples dropped by 27%, 38%, 40%, 41%, 42%, 42%, 42% in 1–7 days, respectively. Thus, the EDTA-treated specimens reached the endpoint after 5–7 days. The selection of endpoint determination methods is key to the preservation of intact collagen because collagen begins to degrade in acidic solutions once the mineral is completely removed^23^. For the weight loss method, demineralization is complete when weight loss stops. Failure to stop the HCl/EDTA mixture treatment as soon as sample transparency was reached resulted in shrunken samples and possibly degraded collagen.

### Raman spectroscopy

**Figure 1:**
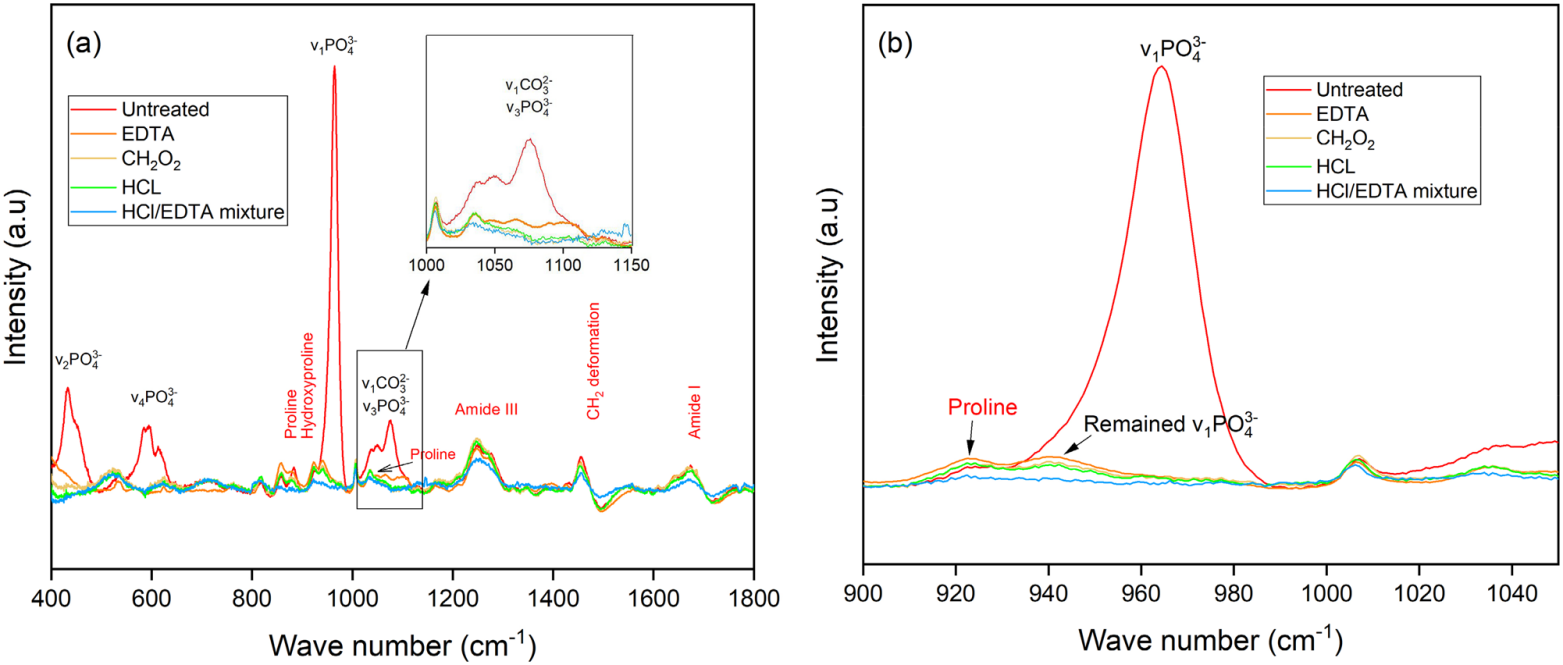
**(a)** Representative Raman spectra indicate mineral and collagen bands for the untreated bone, and for bone treated with ethylene-diamine tetraacetic acid (EDTA), formic acid (CH_2_O_2_), hydrochloric acid (HCl), and the HCl/EDTA mixture. Top right image is an enlarged figure for the overlapping bands of *v*_3_PO and *v*_1_CO. **(b)** Outlined area in (a) with a solid border is shown with the primary phosphate band.

Functional groups and their corresponding wavelengths have been published for collagen and hydroxyapatite^26,32^. As shown in Fig. 1a, the mineral bands *v*_1_PO(~ 959 cm^−1^), *v*_2_PO (~ 430 cm^−1^), and *v*_4_PO (609 cm^−1^–668 cm^−1^) are mostly eliminated by all four chemical treatments, except for the overlapping bands of *v*_3_PO (~ 1035 cm^−1^ and ~ 1076 cm^−1^) and *v*_1_CO (~ 1070 cm^−1^). The remaining signals detected within that overlapping range are possibly proline *v*(C–C) components (an amino acid with carbon backbone)^32^. The EDTA treatment has the highest intensity for *v*_3_PO and *v*_1_CO bands (enlarged at top right in Fig. 1a). Some weak signals were detected under the *v*_1_PO band at 959 cm^−1^ and under proline band at 940 cm^−1^ where the HCl/EDTA mixture treatment had the smallest intensity (Fig. 1b). These findings indicate that the HCl/EDTA mixture removed most of the mineral but affected collagen structure. The collagen bands of proline (~ 853 cm^−1^), hydroxyproline (~ 872 cm^−1^), amide I (1660 cm^−1^, 1690 cm^−1^), amide III (1242–1340 cm^−1^), and CH_2_ (~ 1446 cm^−1^) are mostly preserved compared to the untreated samples. In contrast to the mineral trend, the HCl/EDTA mixture treatment affects the collagen intensity most compared to the other treatments. The intensities of the bands in the HCl and CH_2_O_2_ treated samples signals are between those of the EDTA and HCl/EDTA mixture treatments. The hydroxyproline collagen band is most affected by demineralization, as the peak intensity is largely reduced. A previous study using a lower HCl concentration and a longer treatment time than the conditions used in this paper still retained the hydroxyproline band^33^. However, other collagen bands were not investigated. Demineralization conditions should be further studied to optimize mineral elimination and collagen retention.

**Figure 2:**
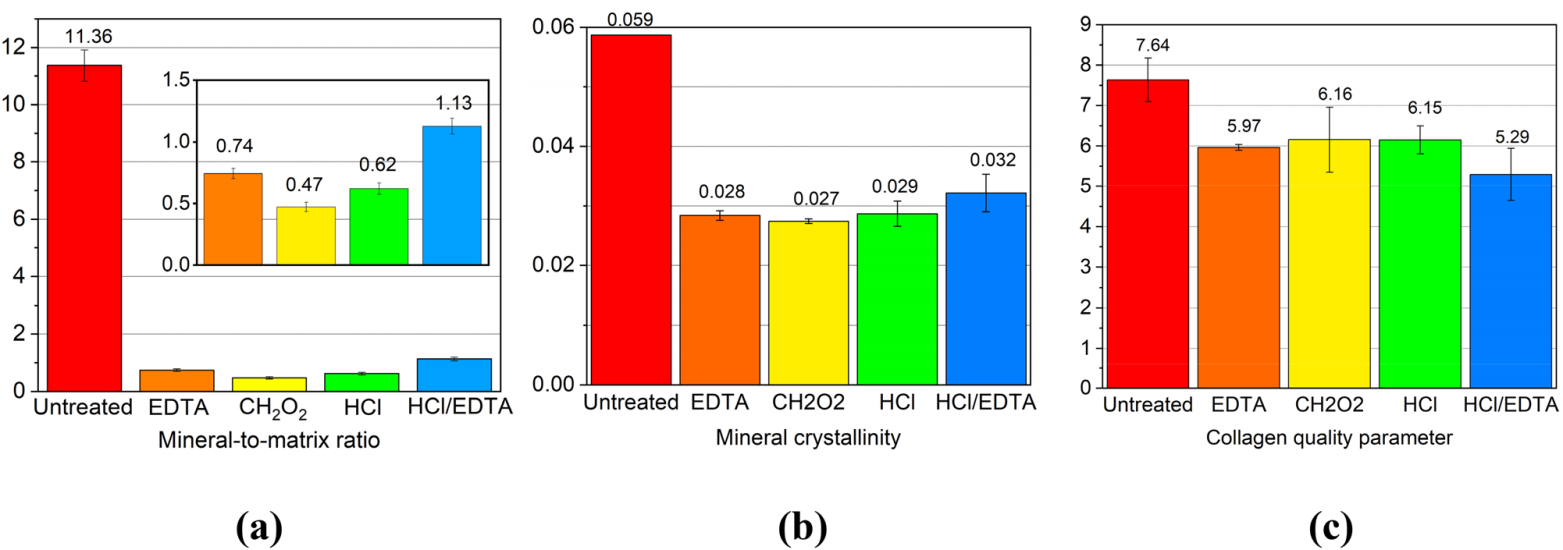
Raman spectroscopy analytical study of the mineral and collagen content for untreated bone, and for ethylene-diamine tetraacetic acid (EDTA), formic acid (CH_2_O_2_), hydrochloric acid (HCl), and the HCl/EDTA mixture treated bone. **(a)** The inset is an enlarged plot of the mineral-to-matrix ratio. Mineral-to-matrix ratio is determined by the intensity ratio of *v*_1_PO_4_^3-^ to amide III bands, **(b)** the mineral crystallinity is defined as 1/FWHM of band *v*_1_PO, and (**c)** the collagen quality is the area ratio of 1660/1690 cm^−1^ under amide I band.

The mineral-to-matrix ratio, mineral crystallinity, and collagen quality were determined for the four types of treated and untreated samples. *p* < 0.05 was for the comparison between untreated bone and each treated bone. *p* > 0.05 was for the comparison between each combination of two different treatments. Figure 2a,b shows that the mineral-to-matrix ratio and mineral crystallinity of CH_2_O_2_-treated bone are smallest. The low crystallinity indicates that the hydroxyapatite changes to a more disordered structure. However, since the signals at 959 cm^−1^ after demineralization are very low, the results on mineral crystallinity (1/FWHM of 959 cm^−1^ band) may not be precise. The HCl/EDTA mixture-treated samples contain the smallest amounts of *v*_1_PO. The high concentration of HCl in the HCl/EDTA solution resulted in faster treatment time but had a strong effect on the remaining collagen. This high concentration led to a smaller amount of mineral and shorter treatment time, which was found in a previous study^21^. However, present results show more changes in the collagen structure from the HCl/EDTA mixture treatment than the EDTA treatment. The HCl/EDTA mixture has the smallest collagen quality parameters as shown in Fig. 2c, which implies the least ordered collagen secondary structure among all treated samples. The evidence is also seen in the amide I band in Fig. 1a. Two peaks at 1660 cm^−1^ and 1690 cm^−1^ are observed in samples from other treatments but only one peak with lower intensity remains in the HCl/EDTA treated samples. Thus, the collagen structure changed most due to the HCl/EDTA treatment. This observation does not agree with the findings of Castania et al*.*^21^. The difference in results may be due to uncertainties in determining the endpoint, and the precision of qualitative (histological analysis with H&E (haematoxylin eosin) staining^21^) and quantitative analyses, such as the sample position in Raman scans. Also, the treated samples may not be demineralized homogeneously. From our results, the collagen components are less affected by the CH_2_O_2_, HCl, and EDTA treatments (Fig. 2c). Among these, the CH_2_O_2_ and HCl most preserve the collagen. Future studies should focus on controlling the concentration of demineralization agents and the reaction endpoint.

Considering the treatment efficiency, HCl takes only seven hours to achieve a similar effect as CH_2_O_2_, which took two days. Acids such as HCl, CH_2_O_2_, and HCl/EDTA mixture (mostly composed of HCl) dissolve the minerals. On the other hand, EDTA removes minerals by binding to calcium ions, resulting in reduced mineral crystal size, which is a diffusion-related process^21^. From this perspective, HCl can be considered the best due to its treatment speed.

### Fourier-transform infrared spectroscopy

**Figure 3:**
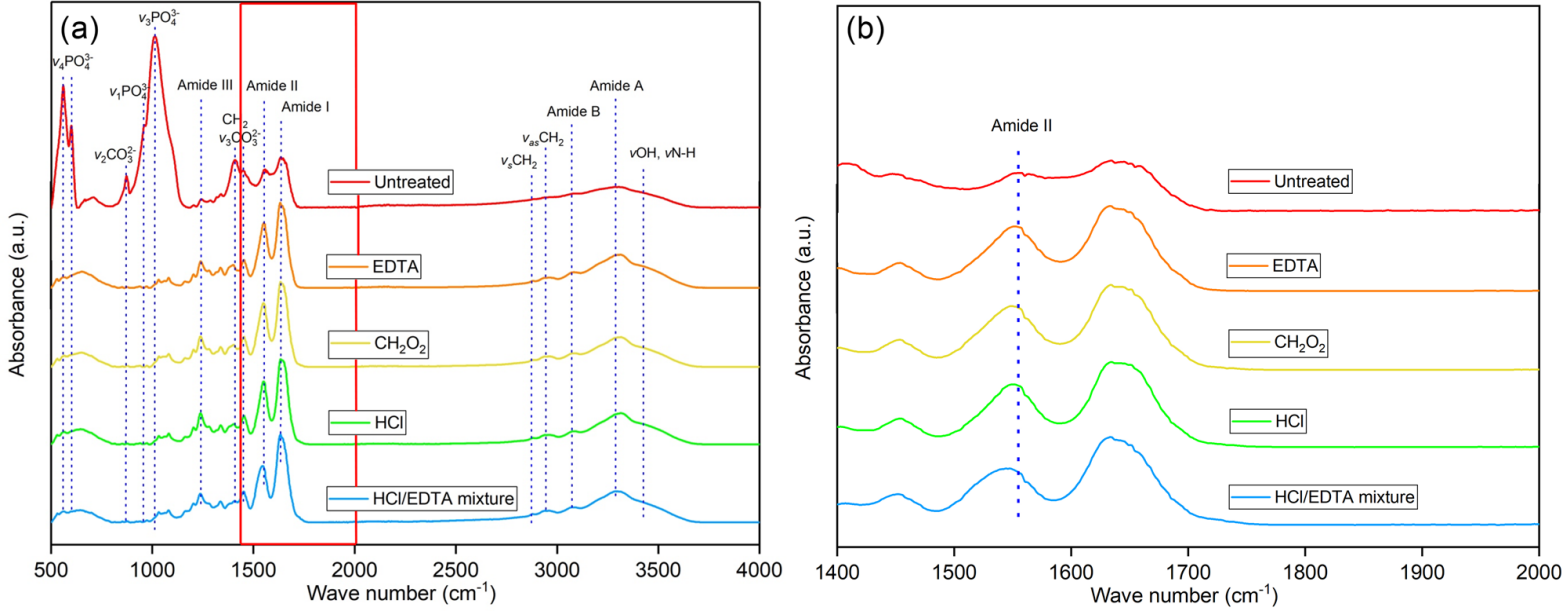
**(a)** Fourier-transform infrared spectra for untreated bone and for ethylene-diamine tetraacetic acid (EDTA), formic acid (CH_2_O_2_), hydrochloric acid (HCl), and the HCl/EDTA mixture treated bone. **(b)** Magnified spectra from the boxed region in (a) showing the shift in the amide II band.

Figure 3 shows the FTIR absorption bands of untreated and treated bones. The bands near 560 cm^−1^ and 600 cm^−1^, 960 cm^−1^, and 1012 cm^−1^ represent a phosphate functional group (PO) with different vibrational modes. The bands around 870 cm^−1^ and 1410 cm^−1^ are from the carbonate functional group (CO). More band assignments are listed in Table 2. The PO and the CO groups are found in untreated bone but disappear in the treated bone. Under the *v*_3_PO band, residual peaks between 1000 and 1100 cm^−1^ are observed in treated samples. The remaining peaks under the band *v*_3_CO near 1410 cm^−1^ are the CH_2_ wag and CH_2_ bend, which are collagen markers^34^.

**Table 2:** Fourier transform infrared spectroscopy absorption bands for bone and their functional groups.

Wave Number (cm^−1^)	Assignments	References
560, 600	*v*_4_${\text{PO}}_{4}^{{3 - }}$	^34,35^
870	*v*_2_${\text{CO}}_{3}^{{2 - }}$	^34,39^
960	*v*_1_${\text{PO}}_{4}^{{3 - }}$	^40,41^
1012	*v*_3_${\text{PO}}_{4}^{{3 - }}$	^34^
1240	Amide III	^42,43^
1410	*v*_3_${\text{CO}}_{3}^{{2 - }}$	^34,35,44^
1555	Amide II	^45^
1634	Amide I	^45^
2850	CH_2_ symmetric stretch	^35^
2930	CH_2_ antisymmetric stretch	^34,35^
3070	Amide B	^46^
3278	Amide A	^34,39^
3407, 3420	*v*OH, *v*NH	^35^

The collagen markers of amide I, II, III, amide A, B, and symmetric and antisymmetric CH_2_ bands are preserved after demineralization, as shown in Fig. 3a. The CH_2_ symmetric (2850 cm^−1^) and antisymmetric stretch bands (2930 cm^−1^) have increased intensities for all treatments because of the increased freedom in vibrational movement of protein due to the breakdown of hydrogen bonds between the hydroxyapatite and protein^35^. The protein secondary structure is a three-dimensional form that consists of repeated hydrogen-bonding with helical and ladder-like patterns^36^. The positions of amide II bands were found to be shifted from 1555 cm^−1^ to a lower wavenumber 1540 cm^−1^ in the HCl/EDTA treated bone (Fig. 3b). This shift in the amide II band can be attributed to the changes in the secondary structure of collagen^37,38^. The shift of the amide II band at 1546 cm^−1^ by around 10 cm^−1^ was observed in a previous study on EDTA treated samples^35^. In this paper, the EDTA, CH_2_O_2_, and HCl treated samples only changed by around 5 cm^−1^, which is much smaller than the shift in HCl/EDTA treated samples (*p* < 0.05 for all other treatments compared to HCl/EDTA mixture). The conclusion that the HCl/EDTA mixture produces the most significant change in collagen structure agrees with the Raman analysis. Amide A and amide B bands were found to have increased intensity after all demineralization. This change leads to more vibrational motion in the protein due to the broken hydrogen bonds between the collagen and the mineral^35^.

Both FTIR and Raman spectroscopies were used in this study to find differences in bone compositions. The collagen secondary structural changes (amide II, and CH_2_ symmetric and antisymmetric stretches) are seen in the FTIR spectra but not in the Raman spectra. In "Raman Spectroscopy", the amide A and B bands are not visible as they are in the FTIR spectra. It is also difficult to identify the overlapping bands of *v*_3_POand *v*_1_CO in Raman, while it is straightforward in FTIR since *v*_3_PO and *v*_1_CO bands are independent and have strong signals. Meanwhile, Raman spectra supplement several mineral and collagen bands such as the *v*_2_PO band, outside of the viewable range of FTIR, and the proline and hydroxyproline bands, which are not easily detectable in FTIR. Most importantly, Raman spectroscopy can quantitatively measure the changes in bone composition after demineralization.

### Thermogravimetric analysis

**Figure 4:**
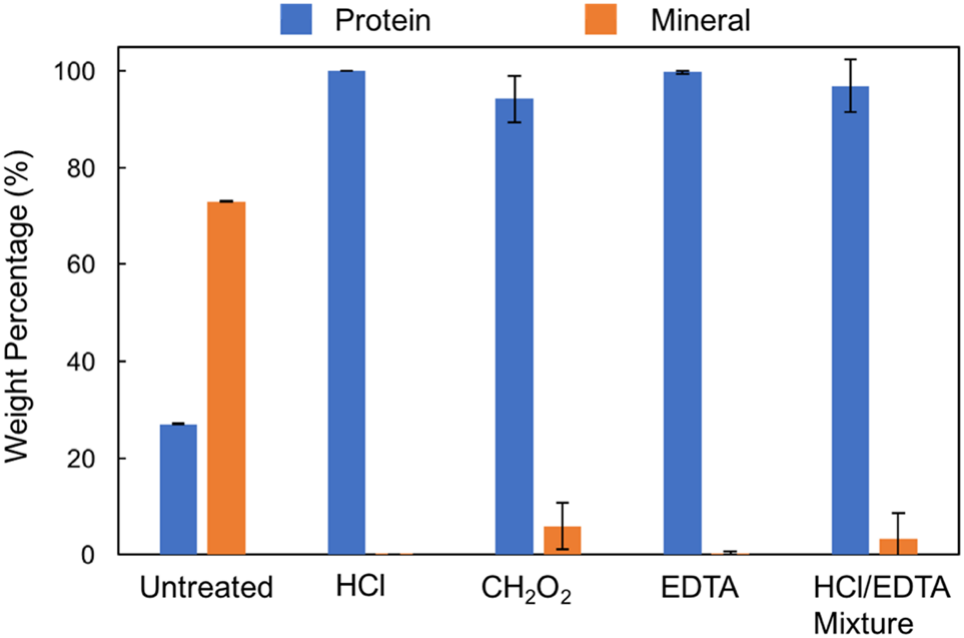
Thermogravimetric analysis results after heating to 800 ˚C: weight percentage of mineral and protein once water is removed after each demineralization treatment.

Figure 4 shows that EDTA and HCl treatments are most effective at removing mineral, while CH_2_O_2_ and HCl/EDTA treatments left ~ 10 wt.% of mineral. This result is at odds with the Raman results, which show that the CH_2_O_2_ treatment removed the mineral while preserving collagen components. The discrepancy between the Raman and TGA results may be because Raman spectroscopy is a surface measurement while TGA measures the composition of the whole bone sample volume. The residual mineral in the samples indicates that the treatment endpoints are difficult to determine using the methods employed in this paper. In particular, the HCl/EDTA mixture endpoint was difficult to gauge due to its high HCl concentration. As a result, there is a delicate balance between the completely demineralizing a sample and over-treating the sample.

### Scanning electron microscopy

**Figure 5:**
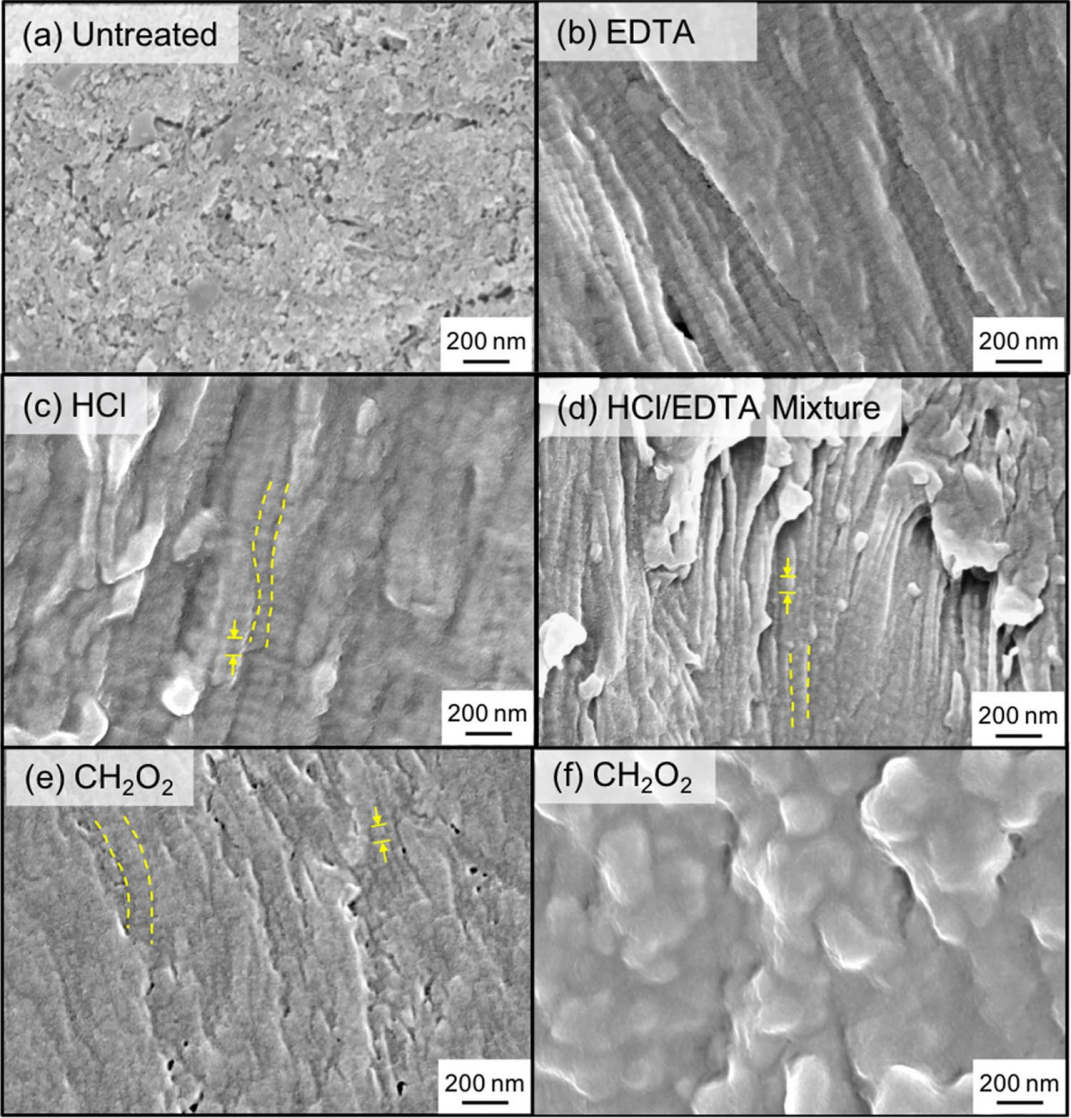
Scanning electron microscopy images of **(a)** untreated bone and demineralized bone using **(b)** ethylene-diamine tetraacetic acid (EDTA), **(c)** hydrochloric acid (HCl), **(d)** HCl/EDTA mixture, and **(e,f)** formic acid (CH_2_O_2_). Visible collagen fibrils are outlined in yellow, dotted lines and *d*-spacing are indicated by arrows.

In untreated bone (Fig. 5a), the main distinguishable feature is the mineral phase. After demineralization, aligned collagen fibrils can be seen in all samples where collagen fibrils are outlined with dotted lines, and the characteristic *d*-spacing of collagen is indicated by arrows. In the EDTA (Fig. 5b), HCl (Fig. 5c), and HCl/EDTA (Fig. 5d) treated specimens, the collagen network is visible, and fibrils are aligned. Samples treated with CH_2_O_2_ have regions where aligned collagen is visible but more difficult to see compared to other samples (Fig. 5e). In the inner cross-section of the CH_2_O_2_ treated samples (Fig. 5f), the collagen structure is not easily visible, which demonstrates that CH_2_O_2_ treated samples are not entirely demineralized, in agreement with TGA results. EDS results (Table 3) show that the weight percentage of phosphate (P) in CH_2_O_2_ treated samples was the smallest. The results agree with Raman’s findings. For all samples excluding the CH_2_O_2_ treated samples, the wt.% of Ca was zero. The mismatch in wt.% calculated between EDS and TGA is likely because EDS is a surface measurement while TGA measures the whole sample mass. Therefore, TGA results are likely more reliable than EDS measurements.

**Table 3:** Elemental analysis from energy dispersive X-ray spectroscopy for untreated specimens, EDTA (ethylene-diamine tetraacetic acid), formic acid (CH_2_O_2_), hydrochloric acid (HCl), and HCl/EDTA mixture treated samples. Both atomic and weight percentages are provided.

Treatment	Untreated			EDTA			HCI			CH 2 0z			HCI/ EDTA		
Element	At.%	Wt.%	*2σ*	At.%	Wt.%	*2σ*	At.%	Wt.%	*2σ*	At.%	Wt.%	*2σ*	At.%	Wt.%	*2σ*
C N O Na P Ca	20.69 13.3 41.6 0.56 9.766 14.07	12.55 9.4 33.6 0.65 15.28 28.48	0. 11 0.2 0.2 0.02 0.05 0.07	60.06 14.8 24. 17 0.238 0.706 0.000	53.71 1 5.4 28.80 0.408 1.628 0.000	0.12 0. 3 0. 1 6 0.017 0.01 6 0.000	60.6 15.85 21 .501 0.414 1.63 0.000	53.8 1 6.39 25.4 0.702 3.74 0.000	0.1 0. 16 0. 11 0.018 0.03 0.000	55.46 1 8.4 25.45 0. 188 0.502 0.004	49.31 19.1 30.15 0.321 1.150 0.013	0.11 0.2 0.16 0.016 0.013 0.007	61.78 13.7 23.62 0. 168 0.736 0.000	55.44 14.3 28.24 0.288 1.703 0.000	0.12 0.3 0.1 6 0.017 0.016 0.000

The relative standard deviations are given as 2$\sigma$.

### Summary: demineralization protocols

The findings from the above experiments revealed that mineral elimination and collagen preservation highly depend on the demineralization agents and that multiple characterization methods are needed to capture these differences. One limitation of this study is that the endpoints vary between treatments and depend on sample size. EDTA samples did not become transparent with treatment, so the transparency method could not be used for EDTA samples. Conversely, the weighing method cannot be used for acid-treated samples because past the endpoint, the acid begins to hydrolyze collagen, meaning that the weight of the sample would continue to decrease even after all mineral is removed. Raman and FTIR analyses of each treated cut sample's interior surface were not done to check if chemicals fully penetrated and reacted with bones.

Thus, future extensions of this work could address more comprehensively the determination of the endpoint of demineralization. This study focuses on how well the mineral content was removed and collagen structure was preserved. Treating time is another important factor that contributes to demineralization efficiency. Future studies could be performed to study mineral ion diffusion as a function of time. The investigation and comparison of the mechanical properties and biocompatibility of the bone demineralized by these four demineralization protocols could also be addressed in a future study.

## Conclusions

Four demineralization agents: ethylene-diamine tetraacetic acid (EDTA), hydrochloric acid (HCl), formic acid (CH_2_O_2_), and ETDA (HCL/EDTA mixture) were used to demineralize bone from six-month-old porcine femurs. The efficacy of mineral removal and the preservation of the collagen structure were compared using qualitative and quantitative methods.

- HCl is proved to be the most efficient demineralization method as it can remove most of the mineral content while preserving collagen structure with short treating time.
- EDTA performs similarly to HCl at removing mineral and preserving collagen integrity, but it takes longer treating time.
- CH_2_O_2_ performed best based on surface measurements but failed to demineralize the inner region of a sample fully. Further studies could be done to see if increasing the treatment time of CH_2_O_2_ would lead to full penetration of samples.
- HCl/EDTA mixture eliminated most mineral content. However, the secondary structure of collagen was also altered.

In summary, this study provides new insights into the efficiency of different bone demineralization methods and sets a framework for future comparative studies. Such knowledge is of high scientific and clinical interest. Fundamental understanding of the structure and composition of bone-derived collagen can guide predictive studies of bone strength and designs of new scaffolds, among other applications.

## Data Availability

Data will be made available upon request.
